# Permanent hypothyroidism following immune checkpoint inhibitors induced thyroiditis may be associated with improved survival: results of an exploratory study

**DOI:** 10.3389/fendo.2023.1169173

**Published:** 2023-04-19

**Authors:** Hanna J. Lee, Anjali Manavalan, Mihaela Stefan-Lifshitz, Clyde Schechter, Aloke Maity, Yaron Tomer

**Affiliations:** ^1^ Division of Endocrinology, Department of Medicine, Albert Einstein College of Medicine, Bronx, NY, United States; ^2^ Department of Family and Social Medicine, Albert Einstein College of Medicine and Montefiore Medical Center, Bronx, NY, United States; ^3^ Department of Medicine, Albert Einstein College of Medicine and Montefiore Medical Center, Bronx, NY, United States; ^4^ Fleischer Institute of Diabetes and Metabolism, Albert Einstein College of Medicine and Montefiore Medical Center, Bronx, NY, United States

**Keywords:** thyroiditis, immune checkpoints, thyrotoxicosis, hypothyroidism, immune checkpoint inhibitors, survival, PET scan, outcomes

## Abstract

**Background:**

Immune-related endocrinopathies are common after immune checkpoint inhibitor (ICI) therapy, among which destructive thyroiditis is the most prevalent. Improved survival outcomes have been associated with immune-related adverse events. We aimed to compare the clinical course and biochemical parameters of two subtypes of ICI-related destructive thyroiditis: a transient thyrotoxicosis that reverts to either euthyroidism (TT; transient thyroiditis) versus progression to permanent hypothyroidism (PH), and to identify prognostic markers in cancer patients receiving ICI therapy who developed DT.

**Methods:**

This retrospective observational study included 124 patients who developed a transient thyrotoxicosis due to a destructive thyroiditis after ICI therapy from January 1, 2016 to April 30, 2021 at the Montefiore Medical Center. Patients were categorized as either TT or PH based on spontaneous renormalization of the TSH or the permanent need for thyroid hormone replacement, respectively. Thyroid hormone and antibody levels, serum inflammatory markers, eosinophils, and metabolic uptake of the thyroid on PET imaging, each corresponding closest to a suppressed TSH, were characterized. Survival from TT and PH were also analyzed.

**Results:**

Of the 124 patients, 53 developed PH and 71 developed TT. The PH group developed thyrotoxicosis at a median of 42 days from the first ICI dose while the TT group took significantly longer at 56 days. Thyroidal PET uptake was increased in 18.9% of the PH group versus 6.0% of the TT group (P=0.04). Three different survival models consistently demonstrated a trend towards increased survival in the PH group, compared to the TT group.

**Conclusion:**

Our results suggest that PH developing after ICI-induced destructive thyroiditis may be associated with a more robust inflammatory and antitumor response to ICI therapy. The results suggests that PH may be a potential clinical predictor of improved survival.

## Introduction

The breakthrough discovery that immune checkpoint molecules are involved in anti-tumor evasion has revolutionized cancer therapy. Currently available immune checkpoint inhibitors are monoclonal antibodies that block negative regulators of the immune system’s anti-tumor effector functions. These specifically target the Programmed cell death-1 (PD-1) receptor expressed on T-cells and its receptor ligand, Programmed-death ligand-1 (PD-L1) expressed by target cells in the tumor microenvironment, as well as the Cytotoxic T-lymphocyte-associated antigen-4 (CTLA-4) found on the cell surface of activated T-cells. Inhibition of these negative regulators allows T-cells to restore and maintain their anti-tumor response (1–3). Immune checkpoint inhibitor (ICI) therapies are now approved for a variety of solid and hematologic malignancies including melanoma, lung cancer, renal cell carcinoma, gastric carcinoma, hepatocellular carcinoma, head and neck squamous cell carcinoma, triple-negative breast cancer, ovarian cancer, Merkel cell carcinoma, and Hodgkin’s and B-cell lymphoma (3, 4).

Due to their immune-stimulatory effects, ICIs can, however, inevitably lead to immune-related adverse events (irAEs) (2). A spectrum of irAEs have been described, affecting many organ systems. Dermatologic, gastrointestinal, pulmonary, and endocrinological manifestations are most common (5, 6). Among the immune related endocrinopathies, thyroid dysfunction is the most prevalent, especially with PD-1 inhibitors, with estimated rates up to 35.5% and more than 50% with combination ICI therapies (2, 7, 8). As a result, the American Society of Clinical Oncology recommends routine monitoring of thyroid function tests during ICI therapy (5). Fortunately, the majority of thyroid adverse events due to ICI are of low-grade toxicities and are often asymptomatic.

Although various patterns of thyroid irAEs can occur, a transient primary thyrotoxicosis that follows the clinical course of a destructive thyroiditis (DT) is most common (9–11). Some patients with ICI-induced DT renormalize back to euthyroidism while others remain permanently hypothyroid. Interestingly, several studies have reported associations of better survival outcomes and progression-free survival with development of immune-mediated thyroid dysfunction, as well as other irAEs, suggesting that thyroid irAEs may be an indirect biomarker of effective treatment-response (6–8, 12).

The aim of our retrospective study was to investigate the incidence, clinical course, and outcomes of CTLA-4, PD-1, and PD-L1 inhibitors-induced thyroid dysfunction among patients at a multi-center urban hospital system that serves an ethnically diverse Bronx population. Unlike previous studies, our primary objective was to compare the clinical behavior and biochemical parameters of ICI-induced thyroid irAEs: severe destructive thyroiditis (DT) resulting in permanent hypothyroidism (PH) versus mild transient thyroiditis (TT).

## Materials and methods

### Study design and participants

This is a retrospective observational study conducted at the Montefiore Medical Center, a health system in the Bronx borough of New York City that includes multiple hospital and outpatient clinical sites. After approval by the Einstein institutional review board, a chart review was conducted for patients who received ICIs from January 1, 2016 to April 30, 2021 and developed transient courses of suppressed TSH (thyroid stimulating hormone) levels. ICIs administered included PD-1 inhibitors (nivolumab, pembrolizumab), PD-L1 inhibitors (atezolizumab, avelumab, durvalumab), and the CTLA-4 inhibitor ipilimumab; a few patients in our cohort were treated with combination anti PD-1 and anti CTLA-4 or anti PD-1 and anti PD-L1 regimens. Only patients with available TSH levels before, during, and after ICI courses were included. Exclusion criteria included age less than 18 years old, pregnancy, pre-existing hypothyroidism or hyperthyroidism, and prior use of thyroid hormone replacement or antithyroid therapies. Measurements of TSH were regularly monitored and typically repeated every 3 to 4 weeks, coinciding with treatment cycles.

### Clinical assessment and definitions

#### Thyroid irAE

In addition to TSH, other thyroid function tests (TFTs), although not available for all patients, were analyzed including free thyroxine (FT4), total thyroxine (TT4), free triiodothyronine (FT3), and total triiodothyronine (TT3). Abnormal TFTs, defined by a suppressed or an elevated TSH, that developed only after initiation of ICI therapy were defined as a thyroid irAE. In this study we focused only on those patients that developed suppressed TSH following ICI therapy. The final cohort of 124 patients were designated as having thyrotoxicosis defined as having a suppressed TSH. Each patient was further subcategorized based on biochemical parameters. Overt hyperthyroidism was defined by a suppressed TSH (our lab reference range RR is 0.4-4.6 uU/mL) with an elevated FT4 (RR 0.8-1.70 ng/dL), if available. In the absence of a FT4, an elevated TT4 (3.2-12.6 ug/dL), FT3 (RR 2.3-4.2 pg/mL), and/or TT3 (RR 90-181 ng/dL) were also defined as overt hyperthyroidism. Subclinical hyperthyroidism was defined by a TSH below the reference interval accompanied by a normal FT4 or, if not available, a TT4, FT3, or a TT3 within the normal reference range. A third category included patients with isolated suppressed TSH levels without any other TFT parameters available. Thyrotoxic clinical symptoms were not a required criteria for thyrotoxicosis.

#### Permanent hypothyroidism and transient thyroiditis with recovery to euthyroidism

All patients in this study experienced a transiently suppressed TSH following ICI therapy and the date of first low TSH defined the onset of the thyroid irAE. PH, following a course of destructive thyroiditis, was defined by an elevated TSH and included both biochemical overt hypothyroidism (high TSH with a low FT4 or, if a FT4 was not available, then with a low TT4, FT3, and/or a TT3) and subclinical hypothyroidism (high TSH with a normal FT4 or, if a FT4 was not available, then with a normal TT4, FT3 and/or TT3). A frankly elevated TSH, in the absence of other available TFT tests, was also included in the definition of PH. Similarly, recovery to euthyroidism, TT, was defined by the eventual development of a normal TSH without need for thyroid hormone replacement.

#### Other parameters

The presence of thyroid antibodies such as the thyroid stimulating immunoglobulin (TSI), thyrotropin binding inhibitory immunoglobulin (TBII), thyroid peroxidase (TPO), and thyroglobulin (Tg) antibodies were included in our analyses, but available in only a minority of the cohort.

Elevated Tg levels (RR of 0-60 ng/mL) and increased standard uptake value (SUV) in the thyroid on ^18^FDG PET (18-fluorodeoxyglucose-positron emission tomography) imaging represented markers of local inflammation of the thyroid.

Increased erythrocyte sedimentation rate (ESR; RR of 0-15 mm/h) and C-reactive protein (CRP; RR of 0-0.8 mg/dL) levels represented markers of systemic inflammation. Eosinophil counts (RR 0.1-0.3 k/uL), which were available for all cohort patients, were also evaluated. To optimally correlate the inflammatory parameters to the thyroid irAE, PET scans and eosinophil counts coinciding to the same day of a low TSH were preferentially analyzed. Otherwise, if not available, PET scans and biochemical values within approximately 4 weeks of a suppressed TSH were included.

### Statistical analysis

The aim of the study was to characterize the natural history, incidence, and clinical outcomes of DT presenting as transient hyperthyroidism that either progressed to permanent hypothyroidism (PH) or recovered to euthyroidism (TT) after ICI therapy. In addition, we analyzed if demographics, thyroidal PET uptake, and biochemical measures including thyroid autoimmunity, systemic and thyroidal inflammatory markers, and eosinophil counts are associated with the subsequent clinical course. Demographic and clinical characteristics were collected for all participants. Descriptive data are reported as: (1) mean ± SD for approximately normally distributed continuous variables; (2) median (interquartile range [IQR]: 25^th^ percentile, 75^th^ percentile) for severely skewed continuous variables; and (3) counts (percentages) for categorical variables. Statistical significance was determined by a two-tailed P value < 0.05. Differences for continuous variables were evaluated using two-sample t-test, or for highly skew distributions the Mann-Whitney U-test, for continuous variables. Chi-square tests were used to measure associations between dichotomous and categorical variables.

### Survival analysis

We investigated if there were differences in overall survival between the PH and the TT subsets after an episode of DT. Three Cox proportional hazards models, stratified on cancer site, with type of DT (PH or TT) as the sole explanatory variable were fit to the data. The decision to stratify on cancer site was taken after exploratory analyses suggested that the hazard functions of the lung and non-lung subsets had noticeably different shapes. Due to lung cancer being the most prevalent malignancy and all others occurring in small numbers of patients, cancer site was dichotomized to lung or non-lung malignancies. The proportional hazards assumption was tested using Schonfeld residuals. Estimated survival functions were graphed. All three models used time to event as outcome, with death as failure; survivors were censored at date they were last known to be alive. The three survival models differed in their origin (start of observation) time. For the first model, the date of ICI initiation was designated as the origin time. The second model used the date of onset of the thyroid irAE as the origin time. The third model used the date at which the DT differentiated into PH or TT as the origin time.

A post-hoc power analysis was conducted using PASS 2023 software. Statistical analysis was done using STATA software, version 17.0 MP4 for Windows (Stata Corp, College Station, TX).

## Results

### Study population

**Figure 1:**
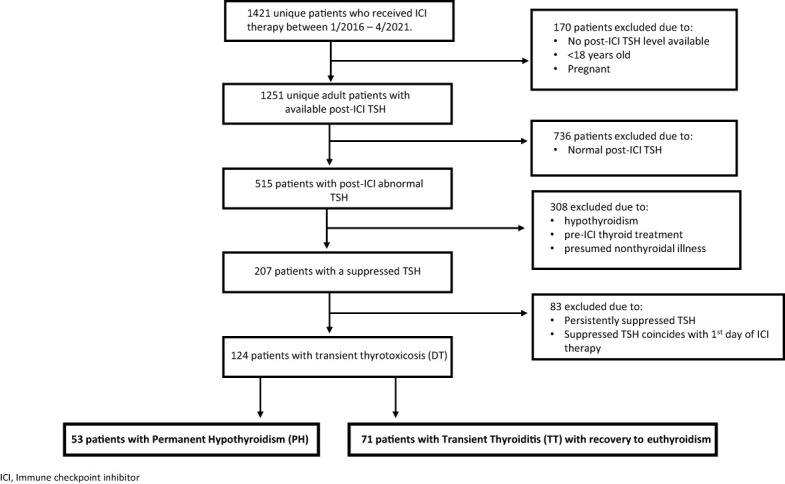
Consort Flow Diagram. ICI, immune checkpoint inhibitor; DT, destructive thyroiditis.

During the study period, we identified a total of 1,421 unique patients who received ICI therapy. 1,251 adult, nonpregnant patients had post-ICI treatment TSH values checked, of whom 515 patients had documented abnormal thyroid function tests, either a low or a high TSH, after ICI initiation. 207 patients specifically developed low TSH levels (thyrotoxicosis) during the observation period. Of these 207 patients, 124 met the inclusion criteria, i.e., they had a transient course of DT in which suppressed TSH levels either inverted to an elevated TSH (Permanent Hypothyroid group; PH) or normalized (transient thyroiditis; TT) (see **Figure 1**). None of the 124 patients had prior exposure to thyroid replacement therapies or anti-thyroid medications nor had a prior history of any thyroid disease.

53 patients (42.7% of the DT patients) developed PH while 71 (57.3%) developed TT. Baseline characteristics including demographic information, primary malignancy, and specific ICI therapies categorized by PH or TT designation are shown in **Table 1**. The PH and TT groups had similar age and sex distributions. There were large differences in the ethnicity distributions of the groups, but ethnicity was not substantially associated with survival (not shown), and so was not treated as a confounding variable.

**Table 1:** Patient characteristics in PH vs TT.

Factor	Overall (124)	PH (53)		TT (71)	
	N	N or mean	% or (s.d.)	N or mean	% or (s.d.)
**Age (years)**		67	**(10.8)**	66	**(11.1)**
Gender					
Male	57	22	**41.5**	35	**49.3**
Female	67	31	**58.5**	36	**50.7**
Ethnicity					
White	41	20	**37.7**	21	**29.6**
AA/Black	47	12	**22.6**	35	**49.3**
Hispanic	17	10	**18.9**	7	**9.9**
Asian	4	2	**3.8**	2	**2.8**
Other	5	4	**7.5**	1	**1.4**
NA/Declined	10	5	**9.4**	5	> **7.0**
Primary Cancer					
Lung	63	19	**35.8**	44	**62.0**
Non-Lung	61	34	**64.2**	27	**38.0**
Non-Lung subtype					
Melanoma	12	5	**9.4**	7	**9.9**
Renal	13	5	**9.4**	8	**11.3**
Breast	4	3	**5.7**	1	**3.2**
Liver	5	5	**9.4**	0	**0**
Endometrial	1	1	**1.9**	0	**0**
Ovarian	1	1	**1.9**	0	**0**
Cervical	3	1	**1.9**	2	**2.8**
Testicular	1	1	**1.9**	0	**0**
Urothelial	1	0	**0**	1	**1.4**
Anal	1	1	**1.9**	0	**0**
Bladder	7	4	**7.5**	3	**4.2**
Prostate	1	1	**1.9**	0	**0**
Lynch Syndrome	2	2	**3.8**	0	**0**
Head & Neck	3	2	**3.8**	1	**1.4**
Esophageal	2	1	**1.9**	1	**1.4**
Colon	2	0	**0**	2	**2.8**
Unknown Primary	2	1	**1.9**	1	**1.4**
Type of ICI					
Pembrolizumab	55	26	**49.1**	29	**40.8**
Nivolumab	40	16	**30.2**	24	**33.8**
Durvalumab	6	3	**5.7**	3	**4.2**
Atezolizumab	13	4	**7.5**	9	**12.7**
Avelumab	3	2	**3.8**	1	**1.4**
Ipilimumab	1	1	**1.9**	0	**0**
Nivolumab + Ipilimumab	3	1	**1.9**	2	**2.8**
Durvalumab + Pembrolizumab	1	0	**0**	1	**1.4**
Nivolumab + Pembrolizumab	2	0	**0**	2	**2.8**
ICI cycles†		12	(16)	12	(17)

PH, permanent hypothyroidism; TT, transient thyroiditis; NA, not available. ^†^median and (interquartile-range). The bold values are referring to the %.

The primary malignancy was lung carcinoma in the majority of patients. 35.8% of the PH group and 62.0% of the TT group were undergoing ICI therapy for a primary lung malignancy. Lung carcinoma was followed, in order of frequency, by melanoma and renal carcinoma. Less frequent malignancies included breast, hepatic, gastrointestinal malignancies, and cancers of the reproductive system, among many others. The PD-1 inhibitors pembrolizumab and nivolumab were the predominant ICI therapies administered. There were no significant differences in the type of ICI regimen nor median number of treatment cycles between the PH and TT groups (**Table 1**).

### Kinetics of DT in PH versus TT

All 124 patients had normal TSH levels prior to ICI initiation. Over a median follow-up duration of 18 months after the first dose, the PH patients developed a suppressed TSH after a median of 42 days from the first ICI cycle while the TT patients took significantly longer at a median of 56 days with a median difference of 14 days (p = 0.03). In the PH group, the median time from documented first ICI dose to a normal TSH, among those with available levels, was 91 days and the median time to the first documented hypothyroidism (elevated TSH) was 105 days. All PH patients, except for three individuals had documented prescriptions for levothyroxine within the Montefiore electronic medical records (EMR) system. The TT patients transitioned to a normal TSH at a median of 145.5 days after the first ICI cycle.

### Biochemical parameters of DT in PH versus TT

Patient charts were reviewed for the presence of the following thyroid antibodies: TPO, Tg antibody, TSI, and TBII. One or more antibodies were checked in 23 PH patients and in 4 TT patients. Among the 23 PH patients, 60.9% (14 patients) demonstrated some degree of autoimmunity with at least a positive TPO and/or Tg antibody; two patients were additionally TBII-positive and one patient was also TSI-positive. Thyroid antibodies were not elevated in the other 9 PH patients. Among the 4 TT patients, all tested negative for the TPO and/or Tg antibodies while two patients also tested negative for TSI. Although only a minor selection of all observed patients had thyroid antibody testing, patients who eventually developed PH manifested a higher prevalence of thyroid autoimmunity compared to those with TT. However, these data need to be confirmed in a larger cohort.

ESR and CRP levels, serum markers of systemic inflammation, were checked in 12 PH and 19 TT patients. Tg levels were checked in 8 PH patients and 4 TT patients. Three patients in each DT subset demonstrated elevated Tg levels; however, the sample size is too small for meaningful analysis. PET scan reports were available for 37 PH patients, among whom 7 patients (18.9%) had increased ^18^FDG uptake at the thyroid. In contrast, of the 67 TT patients with documented PET reports, only 4 patients (6.0%) had increased thyroidal ^18^FDG uptake (p=0.04, **Table 2**). Thus, substantially more PH patients exhibited thyroidal inflammation on PET imaging than the TT patients.

**Table 2A:** Laboratory parameters in PH vs TT.

Factor	Overall (124)	PH (53)		TT (71)	
	N	N	%	N	%
Thyroid Ab (TSI, TBII, TPO, Tg Ab)					
Negative	13	9	**39.1**	4	**100**
≥1 Positive	14	14	**60.9**	0	**0**
Thyroglobulin					
Normal	6	5	**62.5**	1	**25**
Increased	6	3	**37.5**	3	**75**
Inflammatory Markers: ESR, CRP					
Normal	9	2	**16.7**	6	**35.0**
≥1 increased	23	10	**83.3**	13	**65.0**

PH, permanent hypothyroidism; TT, transien thyroiditis. The bold values are referring to the %.

Eosinophils were regularly measured in all patients throughout the observation period. Most eosinophil levels were measured on the same day as the suppressed TSH. 49 of the PH patients (92.5%) had normal eosinophil counts compared to 44 (62.0%) of the TT patients. This was largely driven by a high frequency of eosinopenia (29.6%) in the TT group (**Table 2**). Thus, eosinophil counts were higher (albeit within the normal range) in the PH group compared to the TT group (p = <0.0005).

**Table 2B:** Clinical parameters in PH vs TT.

Factor	Overall (124)	PH (53)		TT (71)		p-value
	N	N	% or (iqr)	N	%	
**Days to 1st suppressed TSH^†^**		42	(50)	56	(77)	0.03*
Thyroid Uptake on PET						
Normal	93	30	**81.1**	63	**94.0**	0.04*
Increased	11	7	**18.9**	4	**6.0**	
Eosinophils						
Normal	93	49	**92.5**	44	**62.0**	<0.0005*
Increased	10	4	**7.5**	6	**8.5**	
Decreased	21	0	**0**	21	**29.6**	

PH, permanent hypothyroidism; TT, transient thyroiditis. *p-value < 0.05. †median and (interquartile-range). The bold values are referring to the %.

### Survival outcomes in DT to PH versus TT

Medical records within the Montefiore Health System, Montefiore’s Tumor Registry, Care Everywhere (the nation-wide EMR integration system), Bronx RHIO (Regional Health Information Organization, a Bronx-specific clinical data exchange) as well as the Social Security Data Exchange were reviewed to determine if patients were alive after study entry. All but two patients, one in each PH and TT subgroup, had discrete documentation of date of death or survival at the conclusion of the study; these two patients were excluded from the survival analyses.

**Figure 2:**
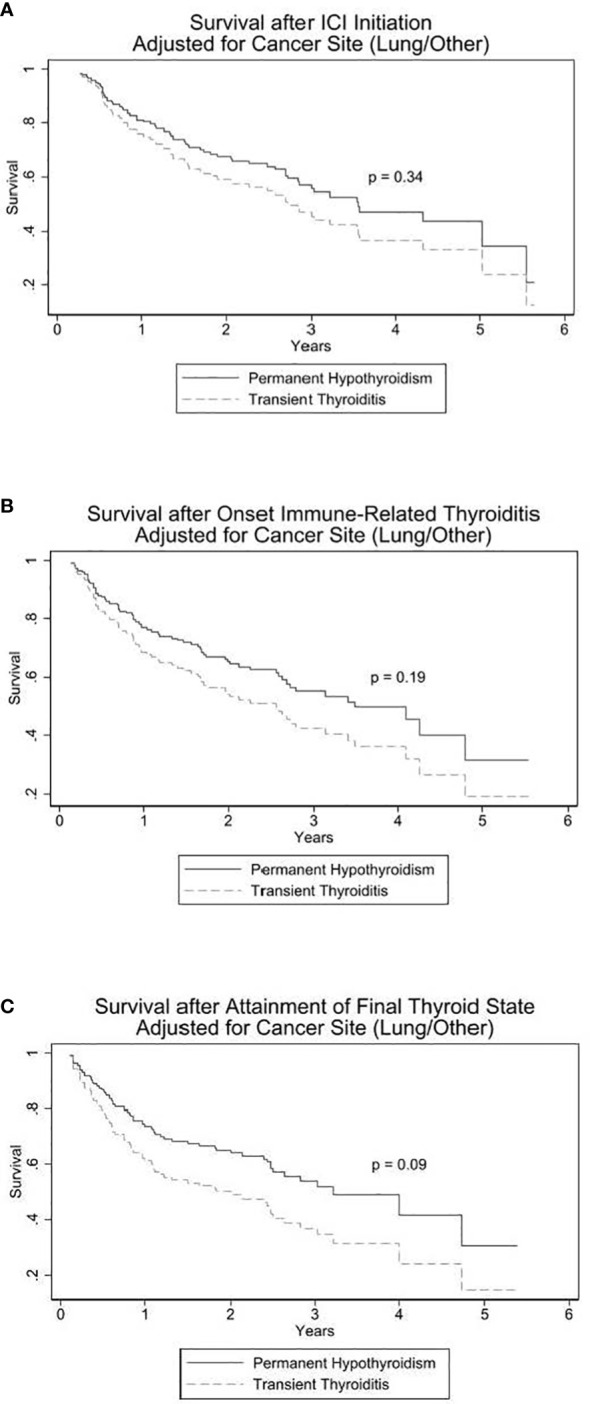
Cox proportional hazards models for survival in PH vs TT. In **(A)**, origin (start of observation) time is date of immune checkpoint inhibitor (ICI) initiation. In **(B)**, origin time is onset of thyroid irAE. In **(C)**, origin time is date at which the DT differentiated into PH (permanent hypothyroidism) or TT (transient thyroiditis).

Three Cox proportional hazards models, stratified by lung versus non-lung primary malignancy, were utilized for survival analysis as described in the Statistical Analysis section. The three models differed in the start-time of follow-up: date of ICI initiation, date of DT onset with suppressed TSH, or from date of PH versus TT development. The TT: PH Hazard Ratios (HR) for death were 1.3 [95% CI 0.8, 2.3], 1.4 [CI 0.8, 2.5], and 1.6 [0.9, 2.8], respectively. Although not statistically significant, each of the three models exhibit a trend toward increased mortality among patients with TT as depicted in **Figure 2**.

Given the patient sample size, number of deaths, and allocation of the sample between PH and TT, the minimum hazard ratio detectable at the 0.05 significance level with 80% power is 2.0 and for 90% power is 2.3.

## Discussion

Immune checkpoint molecules (e.g. CTLA-4, PD-1) keep immune responses in check and play an important role in immune regulation and maintenance of immunological self-tolerance. Blocking immune checkpoint proteins with monoclonal antibodies has emerged as a potent cancer immunotherapy, improving the prognosis for a growing list of advanced malignancies (1, 5). Mechanistically, CTLA-4, PD-1, and PD-L1 inhibitors enhance T-cell proliferation and activation against malignant antigens; the T-cells are reinvigorated to recognize cancer cells, impeding tumor evasion from the immune system. The massive T-cell stimulation induced by ICI can, however, lead to an immune response against self-organs such as the thyroid. There is wide variation in the reported frequencies of thyroid irAEs, likely reflecting differences in study methodologies, biochemical definitions of thyroid dysfunction, as well as patient sample size (2, 3, 5, 8, 11, 13). A common thread across all studies, however, is that ICI-induced thyroid abnormalities are the most common endocrine irAE and manifest early in the treatment course at 5-36 weeks (4). A meta-analysis of 38 prospective controlled trials reported an overall incidence of thyrotoxicosis and hypothyroidism of 2.9% and 6.6%, respectively (2). Observational reports, however, have noted higher frequencies of 7-21% with some studies reporting thyroid dysfunction in as many as 56% of patients on ICI therapy (3, 8, 9, 14, 15). Primary hypothyroidism accounts for up to 31% of the thyroid irAEs and primary hyperthyroidism has been reported to account for up to 46% (3, 9, 11). Of the different etiologies of hyperthyroidism, post-ICI transient thyrotoxicosis due to destructive thyroiditis (DT) has consistently predominated (4, 16, 17). Our study is the first to specifically focus on the biochemical parameters, clinical course, and prognostic outcomes of two key subtypes of DT due to ICI therapy: inversion to permanent hypothyroidism (PH) versus normalization to euthyroidism after a transient thyroiditis (TT).

In our cohort of 1,251 unique patients treated with ICI, an abnormal TSH was noted in 515 patients (41.2%). Among these 515 patients, 178 patients (34.6%) developed a suppressed TSH after the first cycle of ICI therapy of whom 69.7% (124/178) experienced a course of transient hyperthyroidism, classified in our study as DT. The frequencies of an abnormal TSH and of a suppressed TSH in our study are consistent with previously reported rates. Of the patients with DT, 42.7% (53/124) inverted to an overtly elevated TSH; 50 (94.3%) of these 53 patients had documented prescriptions for thyroid hormone replacement. 57.3% (71/124) of patients with DT spontaneously normalized their TSH levels (TT).

One study of 1,246 patients described primary thyrotoxicosis as the most common thyroid irAE, affecting 31% (9). 29% of its thyrotoxic patients experienced a classic thyroiditis pattern of hyperthyroidism followed by a hypothyroid phase; the majority (81%) of the thyrotoxic patients returned to a biochemical euthyroid state. Due to the limited number of studies that specifically analyzed for DT, most involving less than 12 patients with transient thyrotoxicosis (3, 8–10, 12–14, 18), accurate cross-study comparisons cannot be performed. For example, one retrospective study noted that 3 of 7 patients with DT developed PH while another reported that 9 out of 12 thyrotoxic patients eventually transitioned to hypothyroidism. The general trends, however, indicate that a post-ICI DT is frequently encountered (19, 20). The higher occurrence of thyrotoxicosis in our group may be explained by our definition of thyrotoxicosis in which all suppressed TSH levels after the first dose of ICI therapy were included. Of these, 27 patients (21.6%) were overtly thyrotoxic, 62 patients (49.6%) had subclinical thyrotoxicosis, and 36 patients (28.8%) had only suppressed TSH levels available.

Different temporal courses of the two subtypes of DT were observed. The median onset of thyrotoxicosis was earlier at 42 days for the PH subtype after which permanent hypothyroidism developed after another median of 63 days. In contrast, the median onset of thyrotoxicosis was delayed at 56 days for the TT subtype with renormalization of the TSH after a subsequent median of 89.5 days. None of the patients required antithyroid therapy, presumably due to low-grade clinical thyrotoxicosis. Classically, DT (e.g., due to viral infections) transitions through a triphasic course in which the first thyrotoxic phase resolves after 3-6 weeks (21). Approximately a third of patients then enter a hypothyroid phase that can persist up to 6 months. The majority revert to euthyroidism within a year although up to 15% remain permanently hypothyroid. It is possible that some of our study patients categorized as PH are transitioning through a transient hypothyroid phase. However, all PH patients still required levothyroxine replacement therapy more than one year after its initiation, strongly suggesting that they developed permanent hypothyroidism.

The mechanism of ICI-associated thyroid irAEs remains unclear, but the prevailing view is that this is due to an autoimmune lymphocytic destruction of the thyroid (9, 22). However, there is strong evidence that points the underlying mechanism of ICI-associated thyrotoxicosis away from autoimmunity (6, 8, 13, 23). Based on the typical transient thyrotoxic course that spontaneously remits to either hypothyroidism or euthyroidism, post-ICI thyrotoxicosis is likely an inflammatory, destructive process. Moreover, the clinical presentation of rapid destruction of the thyroid observed in ICI-thyroiditis is markedly different than the slow, gradual destruction of the thyroid over many years observed in Hashimoto’s thyroiditis. Furthermore, many patients with ICI-thyroiditis do not have detectable thyroid antibodies such as TRAbs (TSH Receptor antibodies) and do not show signs of autoimmunity. Although some studies have shown baseline TPO and Tg antibody positivity to be more prominent in those who initially present with hypothyroidism and others have shown that these individuals require higher doses of levothyroxine, it is possible that the presence of TPO antibodies may simply reflect a humoral response to exposed thyroid antigens during a destructive process (8, 13) rather than autoimmunity.

Therefore, it is possible that other, thyroid specific mechanisms may contribute to the destruction of the thyroid. Indeed, inflammatory cytokines may contribute to the rapid destruction of the thyroid and the development of DT by direct effects on thyrocytes as we have shown for IFNα-induced thyroiditis (24, 25). Another potential mechanism may be directly mediated by thyroidal PD-L1. PD-L1 expression in thyroid cells has recently been shown by several studies (26–29) and it has been proposed that the inflammatory induction of thyroidal PD-L1 expression (e.g., by IFNγ) is a means of peripheral tolerance to limit self-reactive T-cells (29). PD-1/PD-L1 blockade in the thyroid therefore could lead to the release of self-reactive T-cells under inflammatory conditions. Additionally, in an inflammatory environment, PD-L1 could activate non-immune intrinsic pro-survival signals in thyroid cells, a mechanism demonstrated in cancer cells (30, 31). Thus, the direct inhibition of thyroid-expressed PD-L1 may play a role in thyroiditis development, similar to the role played by pituitary-expressed CTLA-4 in hypophysitis secondary to anti-CTLA-4 therapy (32). Supporting this model, PD-1/PD-L1 therapies are more often associated with development of thyroiditis, while anti-CTLA-4 antibody therapy is more frequently associated with hypophysitis (33, 34).

In our study, more PH patients tested positive for thyroid antibodies. Nevertheless, it is important to note that only a minority of patients had available thyroid antibodies; in fact, 23 patients in the PH group versus only 4 patients in the TT group had levels checked. Furthermore, up to 25% of patients with DT can have low concentrations of thyroid antibodies (21). ESR, CRP, and Tg levels were analyzed as markers of systemic and thyroidal inflammation. Although more PH and TT patients than not had elevated ESR and CRP levels, implying a destructive inflammatory process, conclusive associations are limited due to the few available levels measured in only a few of the patients. These elevated levels may also represent the nonspecific inflammation and chronic stress of an underlying, advanced malignancy. In our cohort, 83% of the patients had PET imaging completed that approximated with the thyrotoxic phase. Increased thyroidal PET-uptake can correlate with or precede thyroidal irAEs, particularly overt biochemical abnormalities, and may serve as both a visual marker and predictor of thyroid dysfunction (8, 10, 14, 20, 35). In our study, more PH patients than TT patients (18.9% PH versus 6.0% TT; p = 0.04) demonstrated diffuse increased ^18^FDG uptake by the thyroid. This suggests that there was significantly more severe inflammation of the thyroid gland in patients who eventually progressed to PH.

An unexpected finding was the significant decrease in eosinophils in the TT group. There is some evidence suggesting that the eosinophil count and eosinophil/monocyte indices can serve as an alternative marker to differentiate between DT and autoimmune hyperthyroidism (36, 37). More specifically, an elevated eosinophil level is associated with Graves’ disease while a low eosinophil/monocyte index ratio suggests DT. For this reason, the eosinophil levels were analyzed and, surprisingly, revealed that 29.6% of TT patients had an eosinopenia, matched to the first suppressed TSH, while none in the PH group had reduced eosinophils. Although the explanation for this trend is not clear, only ~8% of both DT variants had elevated eosinophils which further argues for a destructive thyrotoxicosis rather than an autoimmune process. Another unanticipated finding was the significant difference in the ethnicities between the DT subtypes. Approximately half (49.3%) of the TT patients self-identified as African American/Black versus 22.6% of PH patients. Self-identified White patients accounted for 37.7% and 29.6% in the PH and TT groups, respectively. Hispanic American patients were the third most prominent ethnicity with more represented among the PH patients (18.9% versus 9.9% among the TT patients). A strength of our study, in contrast to most prior publications, is the diversity in ethnicities included [3,8]. However, given the paucity of data, more research is needed to determine if there is a link between ethnicity and a particular DT course.

Our data strongly suggest that the underlying pathogenesis of ICI-induced thyrotoxicosis is primarily a destructive, inflammatory process of the thyroid gland. This ICI-associated inflammatory reaction may be intensified in DT patients who eventually develop PH. Indeed, more PH patients had increased ^18^FDG uptake on PET imaging and experienced an earlier rapid onset of thyrotoxicosis. Moreover, additional factors including genetic susceptibility to autoimmune thyroiditis may contribute to the progression to PH. Indeed, a high polygenic risk score for hypothyroidism was associated with the increased risk of atezolizumab-induced thyroiditis (38).

Interestingly, there was a consistent trend toward increased survival among the PH patients. Although the HRs in the survival analyses are not statistically significant, each survival analysis model (**Figure 2**) demonstrates a shared pattern of elevated mortality in the TT group. This is most prominently displayed in the second and third analytical models in which survival was measured from the onset of DT (p = 0.19; **Figure 2B**) and from the onset of the development of PH or TT (p = 0.09; **Figure 2C**), respectively. The PT and TT survival curves diverge within the first year and remain separate for the remainder of the observation period. Of the three models, the third model not only has an HR closest to reaching statistical significance but, clinically, it has the most predictive applicability in that it suggests that the finite event of PH, compared to TT, following ICI-induced DT may signal longer survival.

Most, but not all studies have also demonstrated that irAEs improve median survival (7, 8, 10, 12, 20, 39–45). For example, one retrospective study of 205 patients calculated the mortality risk as more than double in patients who did not experience a thyroidal irAE (HR 2.43) (6). Likewise, another study of 91 patients reported that patients with post-ICI thyroid dysfunction had a 51% lower mortality (8). It has been suggested that overt thyroid dysfunction may be an even stronger indicator of a better prognosis (35). We hypothesize that the inflammatory and anti-tumor response is amplified in the more severe subtype of DT that eventually transitions to permanent hypothyroidism (PH). ICI therapy induces vigorous activation of the immune system, potentiating cytotoxic T-cell proliferation and cytokine release, likely driving both the desired anti-tumor response and the nonspecific inflammation of bystander organs like the thyroid.

Each of the three survival models has limitations, of which the principal limitation is the small sample size. Indeed, the post-hoc power analysis revealed that an odds ratio of at least 2 is required to have conventional power to find a conventional statistically significant difference, a challenging threshold by any standards. For the first two models, the classification into PH or TT is not known at the origin time of the analysis. The survival differences, particularly those seen with the second and third survival models, may be confounded by immortal time bias in which the TT group took longer from ICI initiation to the first suppressed TSH and likewise took longer to ultimately reach the final phase of euthyroidism. The earlier progression through the DT phases in PH may consequently bias towards its longer subsequent survival outcome. However, the first survival model that counts survival from the first dose of ICI therapy suggested improved overall survival from start of ICI treatment in the PH patients compared to the TT patients. To overcome the immortal time bias, analysis with time-varying covariates was attempted but convergence was not achievable due to the limited number of events and sample size.

Our study has other limitations including those intrinsic to a retrospective observational study. Due to the retrospective nature, not all patients had active thyroid hormone levels tested. Consequently, the definition of thyrotoxicosis was broadened and may have included patients with nonthyroidal illness, potential iatrogenic suppression by iodinated contrast or glucocorticoids as well as, although very rare, central hypothyroidism. Similarly, many patients did not have thyroid antibodies and inflammatory markers consistently checked; we are therefore unable to draw conclusions regarding the potential clinical role of these serological markers. Due to our study’s focus specifically on ICI-induced DT, these results are not generalizable to all thyroid irAE’s following ICI therapy.

Despite the aforementioned limitations, our study is the first known retrospective observational study that specifically focuses on the biochemical characteristics and outcomes of ICI-induced DT and its subtypes of PH and TT in a large number of patients that represent an ethnically diverse patient population. Our study suggests that the PH that follows DT may be a clinical marker of an amplified inflammatory response and enhanced antitumor activity and may be a potential prognosticator of improved survival outcomes. However, because our findings are not definitive, the study will need to be replicated. Further research dissecting the underlying risk factors and mechanisms of ICI-induced thyroid dysfunction are needed to better understand its clinical repercussions.

## Data availability statement

The original contributions presented in the study are included in the article/supplementary material. Further inquiries can be directed to the corresponding author.

## Ethics statement

The studies involving human participants were reviewed and approved by Institutional Review Board of the Albert Einstein College of Medicine. Written informed consent for participation was not required for this study in accordance with the national legislation and the institutional requirements.

## Author contributions

HL, AnM, and YT have made substantial contributions to the concept and design of the study and the analysis of the data. AlM contributed to the data collection. CS performed the statistical analyses and made significant contributions to the study design and analyses. HL, YT, and MSL contributed to the preparation and the drafting of the manuscript. HL, YT, AnM, MSL, and CS reviewed the results and approved the final version of the manuscript.
